# Growth-profile configuration for specific deformations of tubular organs: A study of growth-induced thinning and dilation of the human cervix

**DOI:** 10.1371/journal.pone.0255895

**Published:** 2021-08-11

**Authors:** Kun Gou, Seungik Baek, Marvin M. F. Lutnesky, Hai-Chao Han

**Affiliations:** 1 Department of Mathematical, Physical, and Engineering Sciences, Texas A&M University-San Antonio, San Antonio, Texas, United States of America; 2 Department of Mechanical Engineering, Michigan State University, East Lansing, Michigan, United States of America; 3 Department of Life Sciences, Texas A&M University-San Antonio, San Antonio, Texas, United States of America; 4 Department of Mechanical Engineering, The University of Texas at San Antonio, San Antonio, Texas, United States of America; University of Michigan, UNITED STATES

## Abstract

Growth is a significant factor that results in deformations of tubular organs, and particular deformations associated with growth enable tubular organs to perform certain physiological functions. Configuring growth profiles that achieve particular deformation patterns is critical for analyzing potential pathological conditions and for developing corresponding clinical treatments for tubular organ dysfunctions. However, deformation-targeted growth is rarely studied. In this article, the human cervix during pregnancy is studied as an example to show how cervical thinning and dilation are generated by growth. An advanced hyperelasticity theory called morphoelasticity is employed to model the deformations, and a growth tensor is used to represent growth in three principle directions. The computational results demonstrate that both negative radial growth and positive circumferential growth facilitate thinning and dilation. Modeling such mixed growth represents an advancement beyond commonly used uniform growth inside tissues to study tubular deformations. The results reveal that complex growth may occur inside tissues to achieve certain tubular deformations. Integration of further biochemical and cellular activities that initiate and mediate such complex growth remains to be explored.

## 1 Introduction

Deformations of soft-tissue tubular organs (TOs) are common in human bodies. Tubular organ deformations (TODs) significantly facilitate organ function in the transport of air, fluid, waste, or other materials through the lumens of TOs; typical TODs include, but are not limited to, deformations of blood vessels [1, 2], lymph vessels [3, 4], air ways [5, 6], esophagi [7, 8], human cervices [9, 10], colons [11, 12], and urethrae [13]. Soft tissues are generally considered as hyperelastic materials [14–17]. Various models have been developed to study TODs [18–23] and assist in explaining corresponding deformation-related organ functions in physiopathological conditions. Biological growth means change of mass for organs, which includes increase or reduction of mass that leads to changes in tissue volume or tissue density [24]. Growth is particularly recognized as a significant factor initiating TODs that lead to organ spatial structural responses that are adaptive or pathological [18, 25, 26]. When TODs are initiated partially or fully by growth involving internal volume changes, morphoelasticity is commonly used to illustrate how growth contributes to the total deformation [27–30]. Induced by growth, soft-tissue organs deform themselves as part of normal physiological operations [31], and specific TODs are generated to achieve particular functional needs of the TOs [32]. It is critical to understand how growth is occurring inside the tissue during deformation to more accurately analyze potential pathological conditions and to design effective clinical treatments.

For convenience of use, we define *positive growth* to mean addition of mass that results in an increase of tissue volume [24], and *negative growth* to mean resorption of mass that results in a decrease of tissue volume [33, 34]. It is common to use positive growth in models for organs such as arteries [35] and airways [36], but negative growth is rarely employed in biomechanics to model organ deformation. Furthermore, because of the complexity of tissue composition, different growth may occur inside TOs in different morphometric dimensions to generate needed types of deformations [37]. Such processes can result in counter-intuitive outcomes that more deeply reveal complicated relationships in deformation-targeted growth. To demonstrate such results, we study a particular organ, the human cervix during pregnancy, as a case study to illustrate how special deformations are formed by complex internal growth.

**Fig 1:**
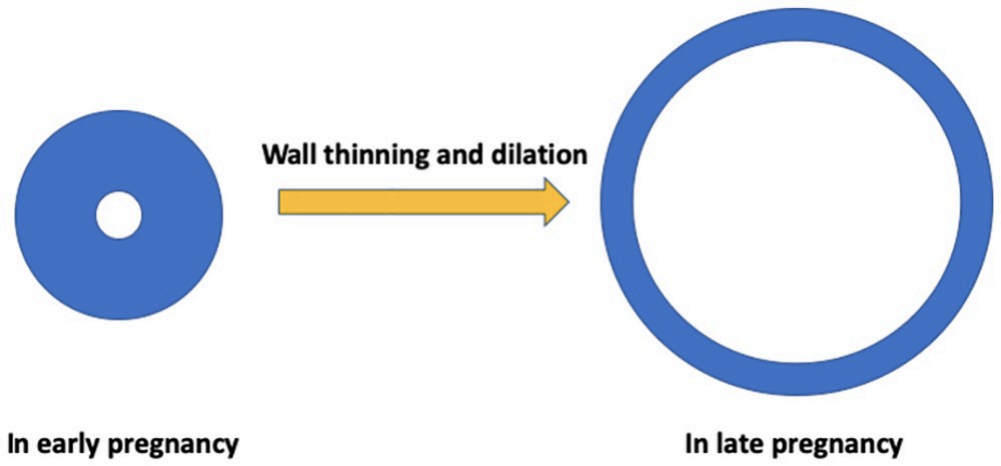
Simple illustration of cervical wall thinning and dilation. Initially the cervix lumen is small and the wall is thick. Gradually the wall becomes thinner and the lumen area increases. Such dimensional changes accommodate the need for a smooth birth.

The cervix is an important reproductive organ below the uterus that keeps the fetus inside the uterus during pregnancy [10, 38, 39]. The cervix remains closed during pregnancy but shows two important deformations including thinning and dilation before the onset of birth (Fig 1) [9, 40–43]. Thinning means the thickness of the cervical wall is reduced, and dilation indicates an enlarged luminal transverse area. The cervical stroma is composed of about 80-85% fibrous connective tissue which is largely responsible for providing the mechanical strength of the cervix; another 10% of the cervical stroma is formed by smooth muscle; while the extracellular matrix consists primarily of collagen, proteoglycans, water, hyaluronan, thrombospondin 2, and elastin [44]. The soft tissue of the cervix is filled with aligned collagen fibers, which gradually become less oriented or diluted leading to reduced fiber stiffness making the cervix softer for smooth birth [45, 46]. The collagen fibers are distributed differently in different parts of the cervix, and the cervix can be approximately differentiated into three layers due to different fiber orientation in each layer [47, 48].

The cervix remodeling/ripening in pregnancy is an important and complex process to prepare for a smooth birth during parturition. This process is initiated and mediated by many biochemical and cellular factors associated with changes to the organ during cervical evolution [45, 47]. For instance, localized regulation of estrogen and progesterone metabolism [49], metalloproteinases, leukocytes, and glycosaminoglycans (GAGs) have been known to play significant roles in initiating the ripening process [50]. Furthermore, leukocytes secrete proteases that can break down the extracellular collagenous matrix and reorganize it to allow for enough cervix dilation and thinning [32], while type I collagen messenger RNA is increased causing collagen synthesis rate to increase. Water increases significantly and dilutes the concentration of the collagen, and noncollagen and nonelastin proteins also increase [51]. Dermatan sulfate concentration is observed to decrease before parturition and bears a possible relation with the expansion of the cervix. Hyaluronic acid (another GAG) concentration increases substantially during the dilation process, and slightly loosens the cervical collagenous network [52]. Smooth muscle cells enlarge, and an increase of smooth muscle may play a great role in rearrangement and orientation of the cervical tissue [51].

In this article, we focus on growth, which is the outcome of all of these biochemical processes or regulations, as an input from a perspective of continuum mechanics without examining the details of these biochemical activities or cellular regulations in the models. The effect of growth is reflected in variation of the growth parameters. Morphoelasticity [24] is used to involve growth in the deformation gradient to achieve the cervix deformations. By testing a range of values of the growth parameters, we summarize how growth in three principle directions collectively generate cervical thinning and dilation. Our study provides an example of exploring deformation-targeted complex growth for TOs.

The structure of the article is as follows. In Sec. 2, we set up the models for the three-layered idealized cylindrical cervix applying morphoelasticity. In Sec. 3, we study how isotropic growth, and growth in each single direction (radial, circumferential, or axial) and their combination contribute to thinning and dilation. Cervical softening by reducing the groundmatrix shear modulus and fiber stiffness is also studied to understand how thinning and dilation can be realized differently. Lastly, in Sec. 4, we summarize and discuss the models and simulations, and how the models can be improved to study more realistic cervical conditions in pregnancy.

## 2 Model setting

**Fig 2:**
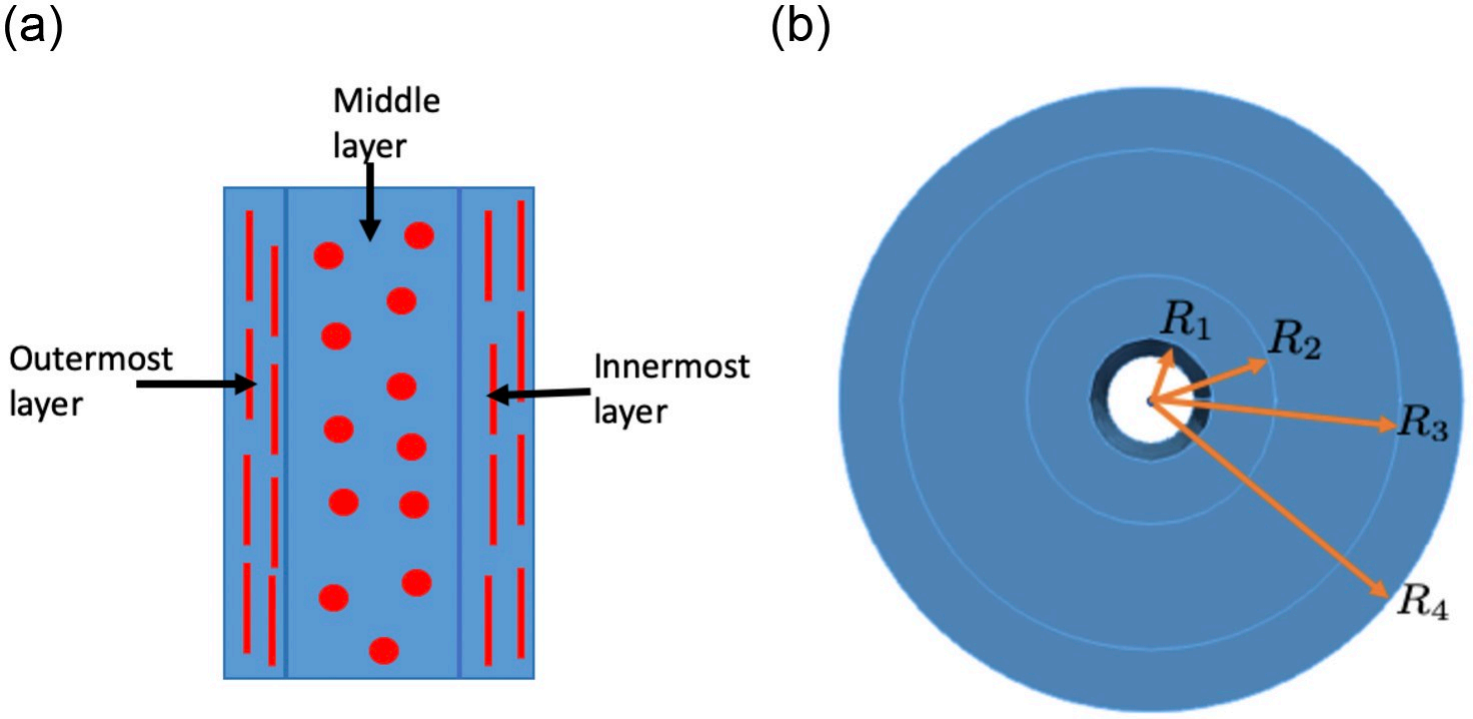
Simple illustrations of the three-layered cervix and its fiber distribution. The left panel (a) illustrates half of an axial cross section of the cervix with different fiber orientations in each layer. The vertical line sections in the innermost and outermost layers illustrate collagen fibers oriented axially, whereas the dots in the middle layer illustrate collagen fibers oriented circumferentially (perpendicular to the axial cross section). The right panel (b) illustrates a transverse section of the cervix with three layers showing the radius prescription for the boundaries and interfaces of the three-layered cervix.

The cervix is roughly cylindrical including three layers with an axially oriented lumen in the middle [47]. For ease of analysis, we idealize the cervix as a regular three-layered cylinder. In the reference configuration (Fig 2b), the radius *R* of the lumen and each layer (the innermost layer, middle layer, or outermost layer) is denoted below

$$\begin{array}{c} \left\{ \begin{array}{cc} \text{Lumen}: & 0<R<R_{1},\\ \text{Innermost}\;\text{layer}\;\text{(Layer}\;\text{1)}: & R_{1}<R<R_{2},\\ \text{Middle}\;\text{layer}\;\text{(Layer}\;\text{2)}: & R_{2}<R<R_{3},\\ \text{Outermost}\;\text{layer}\;\text{(Layer}\;\text{3)}: & R_{3}<R<R_{4}, \end{array}\right. \end{array}$$ (1)

where *R*_*i*_ (*i* = 1, 2, 3, or 4) is the interface or boundary radius.

Any point **X** in the reference configuration, under a deformation mapping **χ**, is mapped to another point **x** in the deformed configuration. The deformation gradient tensor is **F** = ∂**x**/∂**X**. In morphoelasticity, **F** is structured as a product of the growth tensor **F**_*g*_ and the elastic tensor **F**_*e*_ in the form **F** = **F**_*e*_ **F**_*g*_. We consider a cylindrical coordinate system, and the three unit basis vectors in the radial, circumferential, and axial directions are denoted by **e**_*R*_, **e**_Θ_, and **e**_*Z*_, respectively. There is no report of growth difference among these three layers or different parts of the cervix, so not building in differential growth between layers is the simplest first approximation and the most parsimonious assumption. Thus, the growth is taken to be homogeneous within the three layers throughout the cervix. **F**_*g*_ is taken to be a diagonal tensor denoted as

$$\begin{array}{c} F_{g}=g_{r}e_{R}⊗e_{R}+g_{\theta }e_{\Theta }⊗e_{\Theta }+g_{z}e_{Z}⊗e_{Z}, \end{array}$$ (2)

where *g*_*r*_, *g*_*θ*_, and *g*_*z*_ are the growth parameters in the radial, circumferential, and axial directions, respectively. The growth tensor framework in (2), with growth components only in the diagonal, is a commonly used form for other TOs such as blood vessels [18, 25] and airways [26, 36] to study growth-caused deformation in these organs. The elastic deformation is constrained by incompressibility, and thus the elastic tensor **F**_*e*_ satisfies det**F**_*e*_ = 1. The right Cauchy-Green tensor is based only on the elastic deformation part as $C=F_{e}^{\text{T}}F_{e}$, and the three principal invariants based on **C** are *I*_1_ = tr**C**, *I*_2_ = *I*_3_tr**C**^−**1**^, and *I*_3_ = det**C**.

The article [53] showed a photo of the collagen network of the cervix using a harmonic-generation-microscopy imaging technique, and differentiated the cervix into three layers by different collagen orientation. More particularly, in the innermost and outermost layers of the cervix, the collagen fibers are oriented in the longitudinal direction **e**_*Z*_, and in the middle layer, the collagen fibers are oriented in the circumferential direction **e**_Θ_ [48] (Fig 2a). We denote the unit collagen fiber direction vector by **N**_*f*_ satisfying

$$\begin{array}{c} N_{f}=\{\begin{array}{c} e_{Z},\;\text{for}\;R_{1}<R<R_{2}\;\text{or}\;R_{3}<R<R_{4},\\ e_{\Theta },\;\text{for}\;R_{2}<R<R_{3}. \end{array} \end{array}$$ (3)

The fiber-contributed strain energy density function is taken to be [54] $W_{f}=\frac{\gamma }{2}{\left(I_{4}-1\right)}^{2}$, where *γ* is the fiber stiffness parameter, and *I*_4_ is a pseudo-invariant defined by *I*_4_ = **N**_*f*_ · **CN**_*f*_. Such fiber-energy function models general soft tissue, and has been employed in studies for deformations of airways [20, 29].

The strain-energy density function for the isotropic matrix, where the fibers are incorporated, is described by the neo-Hookean model $W_{i}=\frac{\mu }{2}\left(I_{1}-3\right)$, where *μ* is the shear modulus of the matrix. The total energy density of the deformed cervix is *W* = *W*_*i*_ + *W*_*f*_. The Cauchy stress tensor **T** is derived via

$$\begin{array}{c} T=-pI+2F_{e}\frac{\partial W}{\partial C}F_{e}^{\text{T}}, \end{array}$$ (4)

where *p* is an undetermined constraint parameter, and **I** is the identity tensor. **T**_*i*_ is employed to denote the Cauchy stress tensor in the *i*_th_ layer for *i* = 1, 2, or 3. **T**_*irr*_ and **T**_*iθθ*_ are used to express the stress components with respect to **e**_*R*_ ⊗ **e**_*R*_ and **e**_Θ_ ⊗ **e**_Θ_, respectively.

Under growth, the deformation of the cervix is taken to be axisymmetric satisfying

$$\begin{array}{c} x=\chi \left(X\right)=r\left(R\right)e_{R}+Ze_{Z}, \end{array}$$ (5)

where *r*(*R*) is the radial function to exhibit how the radius *R* changes after the deformation. As a first approximation, we assume that the axial position is unchanged by elastic deformation. The boundary or interface radial values *R*_1_, *R*_2_, *R*_3_, and *R*_4_ are mapped to *r*_1_, *r*_2_, *r*_3_, and *r*_4_, respectively, in the deformed configuration.

Our models are based on the assumption of no axial stretch, resulting in fixed top and bottom displacement boundary conditions for the cervix. The purpose is to investigate how growth in each direction determines cervical thinning and dilation without much consideration for other external factors’ influences on the deformations. In practice, the cervix’s top boundary experiences more complicated boundary conditions due to pressure from the fetus and contraction of the uterus [9]. The primary goal of our model is to provide guidance for prescribing more appropriate growth conditions for more realistic models that will eventually encompass major important factors for the cervix in pregnancy. In this article, we also explore how axial stretch contributes to cervical wall thinning and dilation, and make a comparison with results from axial growth to justify the axial stretch selection in (5).

Except for a small amount of mucus [55], no other fluid or air is flowing through the lumen to press the cervical inner boundary, and the outer cervical boundary is also generally free to move under surrounding soft ligaments [56]. Thus for the inner and outer boundaries of the cervix, we consider traction-free boundary conditions. More specifically,

$$\begin{array}{c} T_{1rr}{|}_{R=R_{1}}=0\;\text{and}\;T_{3rr}{|}_{R=R_{4}}=0. \end{array}$$ (6)

At the interfaces of the three layers, the radial components of the Cauchy stress are taken to be continuous satisfying

$$\begin{array}{c} \lim_{r\to r_{2}^{-}}T_{1rr}=\lim_{r\to r_{2}^{+}}T_{2rr},\;\text{and}\;\lim_{r\to r_{3}^{-}}T_{2rr}=\lim_{r\to r_{3}^{+}}T_{3rr}. \end{array}$$ (7)

To obtain the final solution for deformation and Cauchy stress distribution, all the material and geometrical parameters including *R*_1_, *R*_2_, *R*_3_, *R*_4_, *μ*, *γ*, *g*_*r*_, *g*_*θ*_, and *g*_*z*_ are given as input. We need to set up two equations for the unknown *r*_2_ and *r*_3_. The first equation we set up is using (7)_2_ with updated **T**_2_ and **T**_3_, giving

$$\begin{array}{c} T_{2rr}{|}_{r=r_{3}}=T_{3rr}{|}_{r=r_{3}},the\;first\;equation\;for\;r_{2}\;and\;r_{3}. \end{array}$$ (8)

The other equation for *r*_2_ and *r*_3_ is based on continuity for the deformed radius *r*. The radial function *r*(*R*) throughout the cervical wall is

$$\begin{array}{c} r\left(R\right)=\{\begin{array}{c} {\left[r_{2}^{2}-2\int_{R}^{R_{2}}Rg_{r}g_{\theta }g_{z}\text{d}R\right]}^{1/2},\;R_{1}<R<R_{2},\\ {\left[r_{2}^{2}+2\int_{R_{2}}^{R}Rg_{r}g_{\theta }g_{z}\text{d}R\right]}^{1/2},\;R_{2}<R<R_{3},\\ {\left[r_{3}^{2}+2\int_{R_{3}}^{R}Rg_{r}g_{\theta }g_{z}\text{d}R\right]}^{1/2},\;R_{3}<R<R_{4}. \end{array} \end{array}$$ (9)

By (9)_1_ and (9)_2_, *r* is automatically continuous at the interface *R* = *R*_2_. By (9)_2_ and (9)_3_, the continuity of *r* at *R* = *R*_3_ generates

$$\begin{array}{c} {\left[r_{2}^{2}+2\int_{R_{2}}^{R_{3}}Rg_{r}g_{\theta }g_{z}\text{d}R\right]}^{1/2}=r_{3},the\;second\;equation\;for\;r_{2}\;and\;r_{3}. \end{array}$$ (10)

After solving for *r*_2_ and *r*_3_ numerically from (8) and (10), we can obtain the complete *r* as a function of *R*, and also the Cauchy stress tensor **T** to analyze how different growth affects the deformation.

## 3 Computational results

We use biologically-relevant values to parameterize the simulations. In [57], the authors studied eight women whose ages were between 36 and 43 years old and were under hysterectomy with their cervices and uteruses removed due to pathology not related to the cervix. The size of the cervix from each woman was different, and the average of these data is employed as the geometrical parameters of the cervix in this article; more particularly, *R*_1_ = 4 mm, *R*_2_ = 8 mm, *R*_3_ = 16 mm, and *R*_4_ = 20 mm. The cervical length is 50 mm [19, 38], but is unchanged according to the deformation defined in Eq (5). For the stiffness parameters [19, 58], *μ* = 1650 Pa, and *γ* = 1000 Pa (estimated). To elucidate how cervical wall thinning and dilation are associated with the growth parameters *g*_*r*_, *g*_*θ*_, and *g*_*z*_, we study the deformation results for isotropic growth (*g*_*r*_ = *g*_*θ*_ = *g*_*z*_) and anisotropic growth by varying one growth parameter while fixing the other two parameters. We also study how integratively the three parameters with different quantities contribute to the deformation. *Growth with the growth parameter less than one means negative growth, and growth with the growth parameter greater than one means positive growth*. In the literature, there are no clear definitions for cervical thinning and dilation and no standards concerning how to quantify the two deformations. Thinning is easier to understand intuitively, but there are multiple ways to define dilation, such as using inner or outer diameter/radius changes. For instance, in [51], Leppert measured the outer diameter of the cervix to be 10 cm (with the outer radius to be 5 cm) compared with the original outer radius of 2 cm to indicate how extensively the cervix was dilated. Correspondingly, both the inner and outer radii of the cervix increase greatly during pregnancy to prepare the cervical lumen as a large canal for smooth birth. To obtain a strong dilation effect, it is better to use both inner and outer radii to understand dilation. For clarity, we define cervical wall thinning and dilation as follows:

- **Cervical wall thinning**: The cervical wall thickness after growth is less than the original wall thickness, i.e., *r*_4_ − *r*_1_ < *R*_4_ − *R*_1_ = 16 mm.
- **Cervical wall dilation**: After growth, the radius for the inner boundary is greater than the original inner boundary radius, i.e., *r*_1_ > *R*_1_, and the radius for the outer boundary is greater than the original outer boundary radius, i.e., *r*_4_ > *R*_4_.

### 3.1 Isotropic growth

**Fig 3:**
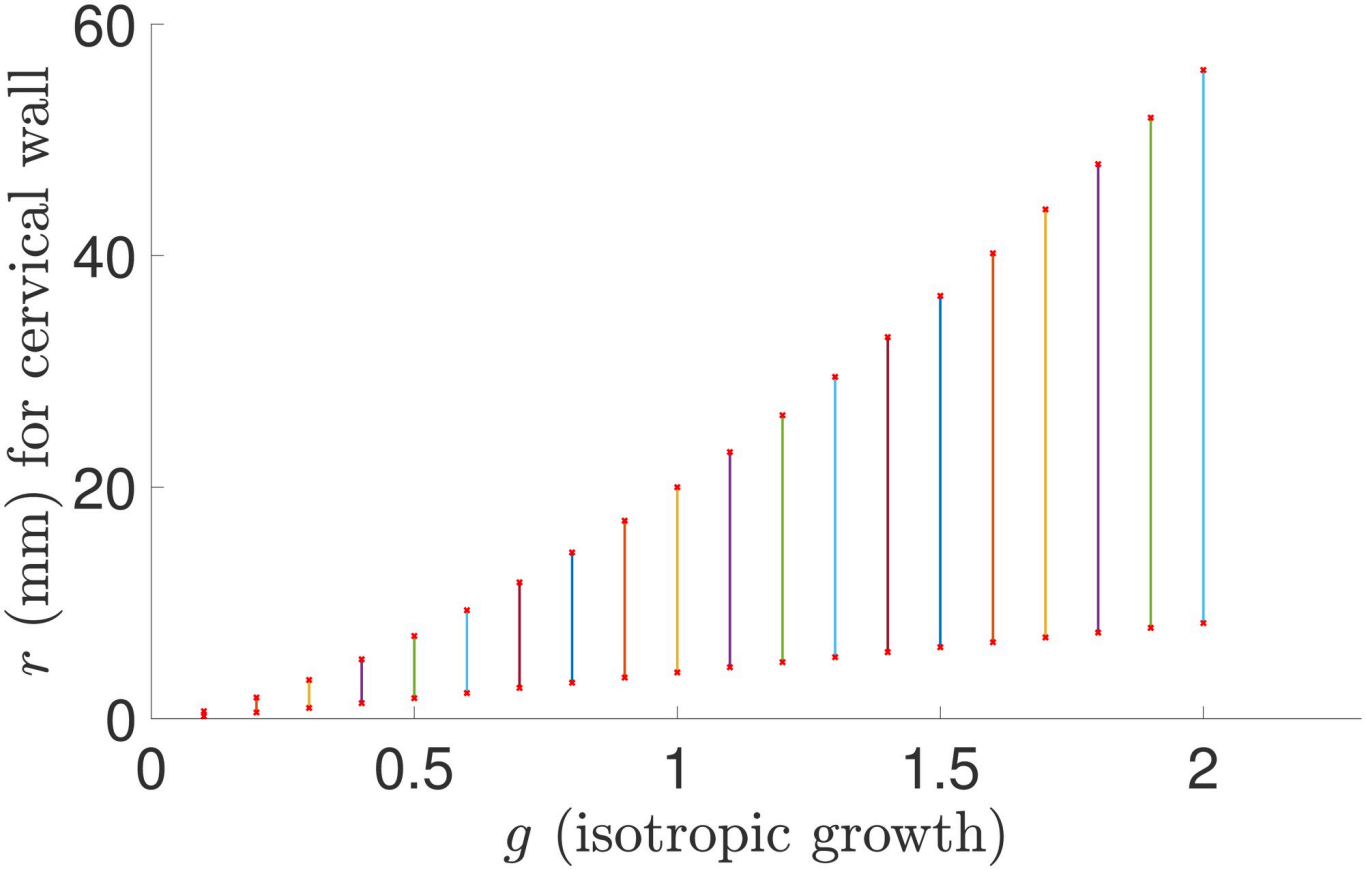
The cervical wall radius and thickness for the isotropic growth parameter *g* over (0.1, 2). The lower point of each line interval shows *r*_1_ for the inner boundary, and the upper point shows *r*_4_ for the outer boundary. The length of each vertical line section illustrates the cervical wall thickness corresponding to a specific radial growth parameter.

We use *g* to represent the isotropic growth parameter satisfying *g* = *g*_*r*_ = *g*_*θ*_ = *g*_*z*_. In the simulations, several parameters including 0.3, 0.7, 1.3, and 1.7 are used for *g* to represent negative or positive growth. The simulation results are shown in Table 1. The results demonstrate that, for negative growth with *g* = 0.3 and 0.7, the wall is thinned but fails to dilate, and that, for positive growth with *g* = 1.3 and 1.7, the wall is not thinned but dilates. We also more vividly illustrate the wall change effect for more values of *g* over the interval (0.1, 2) in Fig 3. The simulation shows the same pattern, i.e, negative growth only makes the cervical wall thinned but does not dilate the wall, and positive growth only dilates the wall, but instead of thinning the wall, thickens the wall greatly due to the rapid volume increase from isotropic growth in all three directions. The outcome suggests that *isotropic growth cannot reach the goal of both thinning and dilation simultaneously*. Anisotropic growth can be considered. In the following three subsections, we evaluate how wall thinning and dilation may occur for growth in each single direction.

**Table 1:** Cervical wall thinning and dilation results for isotropic growth with *g* = *g*_*r*_ = *g*_*θ*_ = *g*_*z*_. Length unit: mm.

*g*	*r*_1_	*r*_4_	Thickness	Thinning	Dilation
0.3	0.9	3.4	2.4	Yes	No
0.7	2.7	11.8	9.1	Yes	No
1.3	5.3	29.5	24.2	No	Yes
1.7	7.0	44.0	37.0	No	Yes

### 3.2 Radial growth

First we employ a few discrete values for the radial growth parameter *g*_*r*_ varying among 0.3, 0.7, 1.3, and 1.7. The growth parameters in the circumferential and axial directions are fixed to be *g*_*θ*_ = 1 and *g*_*z*_ = 1. The outcome is illustrated in Table 2. It shows that negative radial growth generates thinning but not dilation, and positive growth does not generate thinning or dilation.

**Table 2:** Cervical wall thinning and dilation evaluation for different radial growth parameters *g*_*r*_ under *g*_*θ*_ = *g*_*z*_ = 1. Length unit: mm.

*g*_*r*_	*r*_1_	*r*_4_	Thickness	Thinning	Dilation
0.3	9.5	14.3	4.8	Yes	No
0.7	6.1	17.5	11.4	Yes	No
1.3	2.7	22.5	19.8	No	No
1.7	1.9	25.6	23.7	No	No

**Fig 4:**
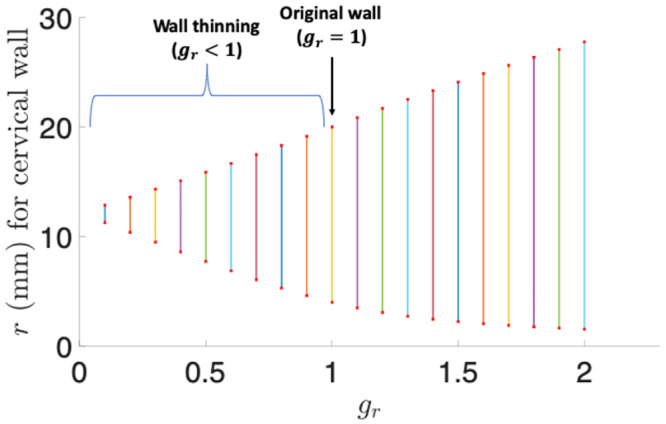
The cervical wall radius and thickness for the radial growth parameter *g*_*r*_ over (0.1, 2). The lower point of each line interval shows *r*_1_ for the inner boundary, and the upper point shows *r*_4_ for the outer boundary. The length of each vertical line section illustrates the cervical wall thickness corresponding to a specific radial growth parameter.

To more clearly demonstrate how the cervical wall thickness and dilation are dependent on radial growth, we use a continuous interval of *g*_*r*_ from 0.1 to 2. When *g*_*r*_ increases, *r*_1_ for the inner boundary decreases monotonically, *r*_4_ for the outer boundary increases monotonically, and the wall thickness (*r*_4_ − *r*_1_) also increases monotonically (Fig 4). For negative growth (*g*_*r*_ < 1), it makes the wall thinner than the original wall with the thickness 16 mm, and smaller *g*_*r*_ generates thinner wall. Positive growth (*g*_*r*_ > 1) thickens the wall. For each *g*_*r*_, either *r*_1_ < *R*_1_, *r*_4_ < *R*_4_, or both occur, and thus none of these *g*_*r*_ show a dilation effect.

### 3.3 Circumferential growth

**Fig 5:**
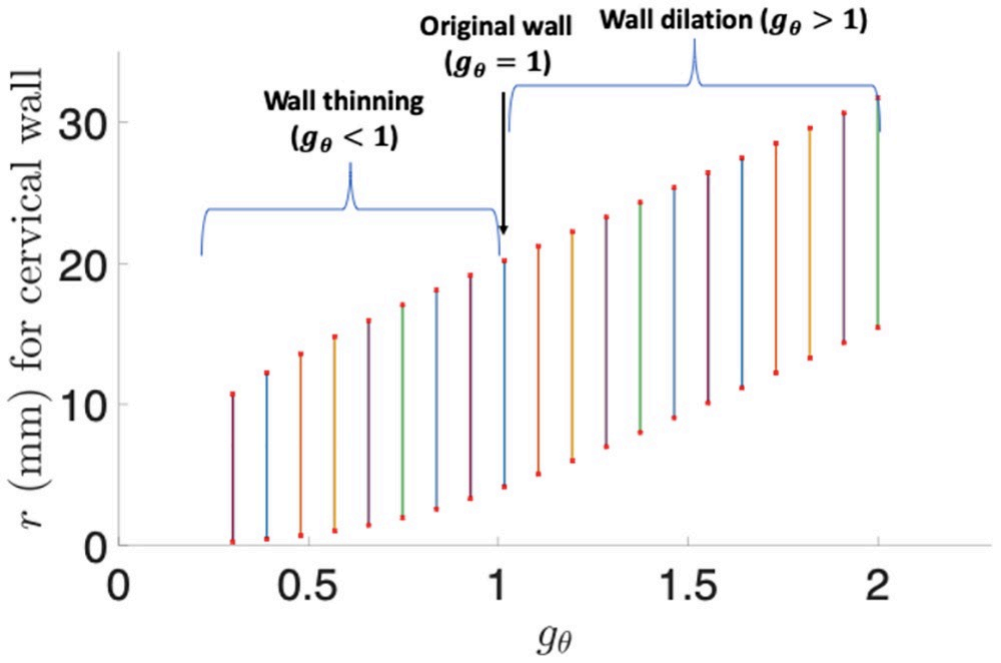
The cervical wall radius and thickness for the circumferential growth parameter *g*_*θ*_ over a continuous interval (0.3, 2). The top points of the vertical lines represent *r*_4_, the bottom points represent *r*_1_, and the length of each vertical line represents the wall thickness.

The same set of discrete parameters for radial growth is employed for circumferential growth, i.e., *g*_*θ*_ = 0.3, 0.7, 1.3, and 1.7, under *g*_*r*_ = *g*_*z*_ = 1. See Table 3 for the results. Negative growth (*g*_*θ*_ = 0.3, 0.7) makes the wall thinned, and positive growth (*g*_*θ*_ = 1.3, 1.7) makes the wall slightly thickened. The negative circumferential growth does not dilate the wall, but positive circumferential growth dilates the wall. Fig 5 illustrates the wall thinning and dilation effect for *g*_*θ*_ over a continuous interval (0.3, 2). The thickness of the wall remains relatively constant for circumferential growth. As *g*_*θ*_ increases, the dilation effect becomes obvious.

**Table 3:** Cervical wall thinning/thickening and dilation results for different circumferential growth parameters *g*_*θ*_ under *g*_*r*_ = *g*_*z*_ = 1. Length unit: mm.

*g*_*θ*_	*r*_1_	*r*_4_	Thickness	Thinning	Dilation
0.3	0.3	10.7	10.5	Yes	No
0.7	1.7	16.5	14.8	Yes	No
1.3	7.2	23.5	16.3	No (slightly)	Yes
1.7	11.9	28.2	16.3	No (slightly)	Yes

### 3.4 Axial growth

**Fig 6:**
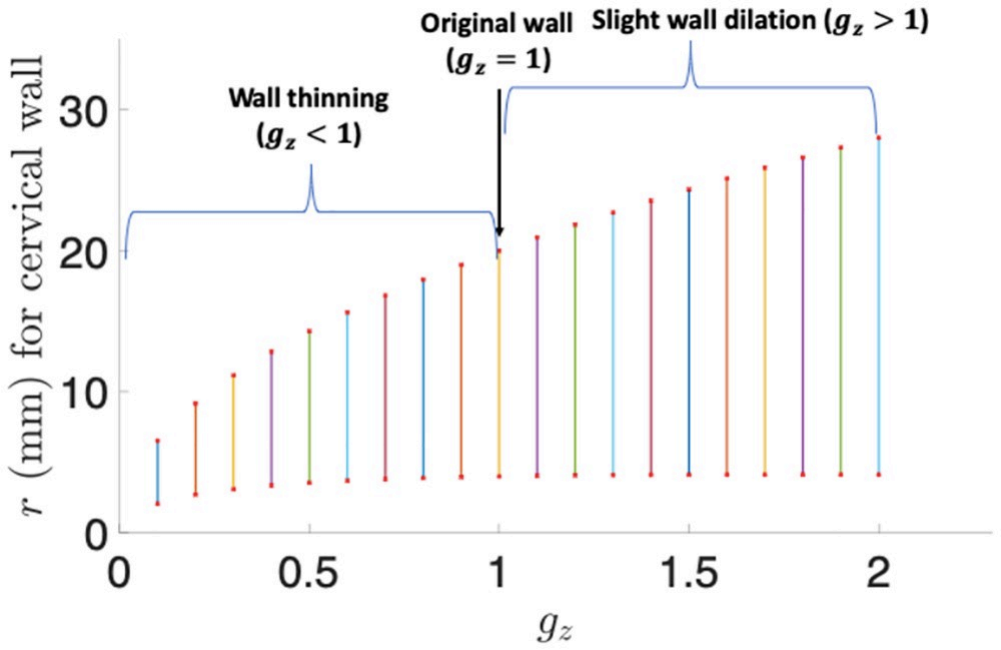
The cervical wall radius and thickness for the axial growth parameter *g*_*z*_ over a continuous interval (0.1, 2). The top pints of the vertical lines represent *r*_4_, the bottom points represent *r*_1_, and the length of each vertical line represents the wall thickness.

Table 4 demonstrates the thickening and dilation effects for axial growth again using four parametric values 0.3, 0.7, 1.3, and 1.7 for *g*_*z*_ under *g*_*r*_ = *g*_*θ*_ = 1. Only when *g*_*z*_ < 1, can the wall be thinned. Dilation occurs only when *g*_*z*_ > 1, but is weak because the inner boundary radius *r*_1_ is only slightly greater than the original radius *R*_1_ = 4 mm even for larger *g*_*z*_ parameters. Fig 6 shows the wall radius and thickness for a continuous interval of *g*_*z*_ in (0.1, 2). The figure more clearly illustrates that the inner boundary *r*_1_ remains almost unchanged, and thus indicates that the axial growth is insufficient to increase the luminal area much. During pregnancy, the cervix is shortened [42], and *g*_*z*_ < 1 can be used to realize cervical shortening. Thus we do not consider axial growth generated dilation.

**Table 4:** Cervical wall thinning and dilation results for different axial growth parameters *g*_*z*_ under *g*_*r*_ = *g*_*θ*_ = 1. Length unit: mm.

*g*_*z*_	*r*_1_	*r*_4_	Thickness	Thinning	Dilation
0.3	3.1	11.2	8.1	Yes	No
0.7	3.8	16.8	13.0	Yes	No
1.3	4.1	22.7	18.6	No	Yes (slightly)
1.7	4.1	25.9	21.8	No	Yes (slightly)

We also study how axial stretch other than axial growth contributes to cervical wall thinning and dilation. If an axial stretch factor λ_*z*_ is considered in the deformation (5), the deformation function is updated to

$$\begin{array}{c} x=\chi \left(X\right)=r\left(R\right)e_{R}+\lambda_{z}Ze_{Z}. \end{array}$$ (11)

**Fig 7:**
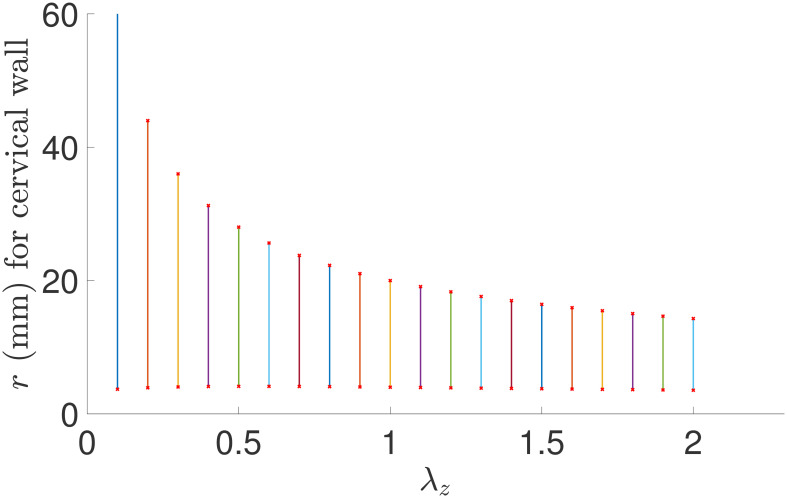
The cervical wall radius and thickness for the axial stretch parameter λ_*z*_ over a continuous interval (0.1, 2). Interpretation of the vertical line intervals are the same as in Fig 6.

Consequently, if the original length of the cervix is *L*, then the length of the deformed cervix becomes λ_*z*_ *L*. Furthermore, if λ_*z*_ < 1, the cervix is shortened, and if λ_*z*_ > 1, the cervix is elongated. We also assume no growth occurs in the cervical tissue, i.e., **F**_*g*_ = **I**; see results in Fig 7. Comparing with results in Fig 6 for *g*_*z*_, Fig 7 shows an opposite direction for thinning and dilation of the cervix. Namely, the largest wall thickness value is obtained at the smallest λ_*z*_ value, and the smallest thickness value occurs at the largest λ_*z*_ value. The thickness increases greatly as λ_*z*_ decreases. By checking the endpoint values of each vertical line section for *r*_1_ and *r*_4_ (data not shown for brevity), as λ_*z*_ < 1, thinning cannot occur and little or no dilation occurs, while as λ_*z*_ > 1, dilation cannot occur but thinning is achieved. The results demonstrate that pure axial growth cannot thin and dilate the cervix simultaneously. Additional growth is required to obtain the two deformations. Considering the cervix is shortened during pregnancy, only λ_*z*_ < 1 should be employed.

To investigate how λ_*z*_ < 1 influences thinning and dilation for different axial growth parameters *g*_*z*_, we work on two pairs of parameters: (1) λ_*z*_ = 0.8 and *g*_*z*_ = 0.7, and (2) λ_*z*_ = 0.8 and *g*_*z*_ = 1.3. Note both (1) and (2) are with *g*_*r*_ = *g*_*θ*_ = 1. For (1), *r*_1_ = 3.9 mm, *r*_4_ = 18.6 mm, and thickness = *r*_4_ − *r*_1_ = 14.7 mm; for (2), *r*_1_ = 4.1 mm, *r*_4_ = 25.3 mm, and thickness = *r*_4_ − *r*_1_ = 21.2 mm. Comparing with results in Table 4 for *g*_*z*_ = 0.7, results for (1) show larger *r*_1_ and *r*_4_ with a thicker wall, and the same pattern appears for (2) under *g*_*z*_ = 1.3. Such outcomes demonstrate that λ_*z*_ < 1 facilitates dilating the cervix but reduces the thinning effect, and that a stronger axial growth effect is required to realize the expected thinning for such λ_*z*_. We take λ_*z*_ = 1 in the deformation mapping in (5) to reduce the possible counter-effect on thinning or dilation from other non-identity axial stretch ratios for a focused study on outcomes from growth effects in the three principle directions.

### 3.5 Combined growth

According to the results from radial, circumferential, and axial growth, we summarize the combined thinning and dilation effects in Table 5. Only *g*_*θ*_ > 1 can generate substantial dilation. All negative growth in the three directions generate thinning effects; *g*_*r*_ < 1 generates the strongest thinning effect, and *g*_*θ*_ < 1 generates the weakest thinning effect. In summary, to realize both thinning and dilation in the deformation, positive circumferential growth and negative radial/axial growth are required. Because negative radial growth generates the strongest thinning effect, it is preferably used to produce thinning effects.

**Table 5:** Summary of different circumstances generating thinning and dilation. Over the row for thinning, the order is made from strong to weak, i.e., *g*_*r*_ < 1 produces the strongest thinning effect, and *g*_*θ*_ < 1 produces the weakest thinning effect.

Thinning:	*g*_*r*_ < 1,	*g*_*z*_ < 1,	or *g*_*θ*_ < 1
Dilation:	*g*_*θ*_ > 1		

**Fig 8:**
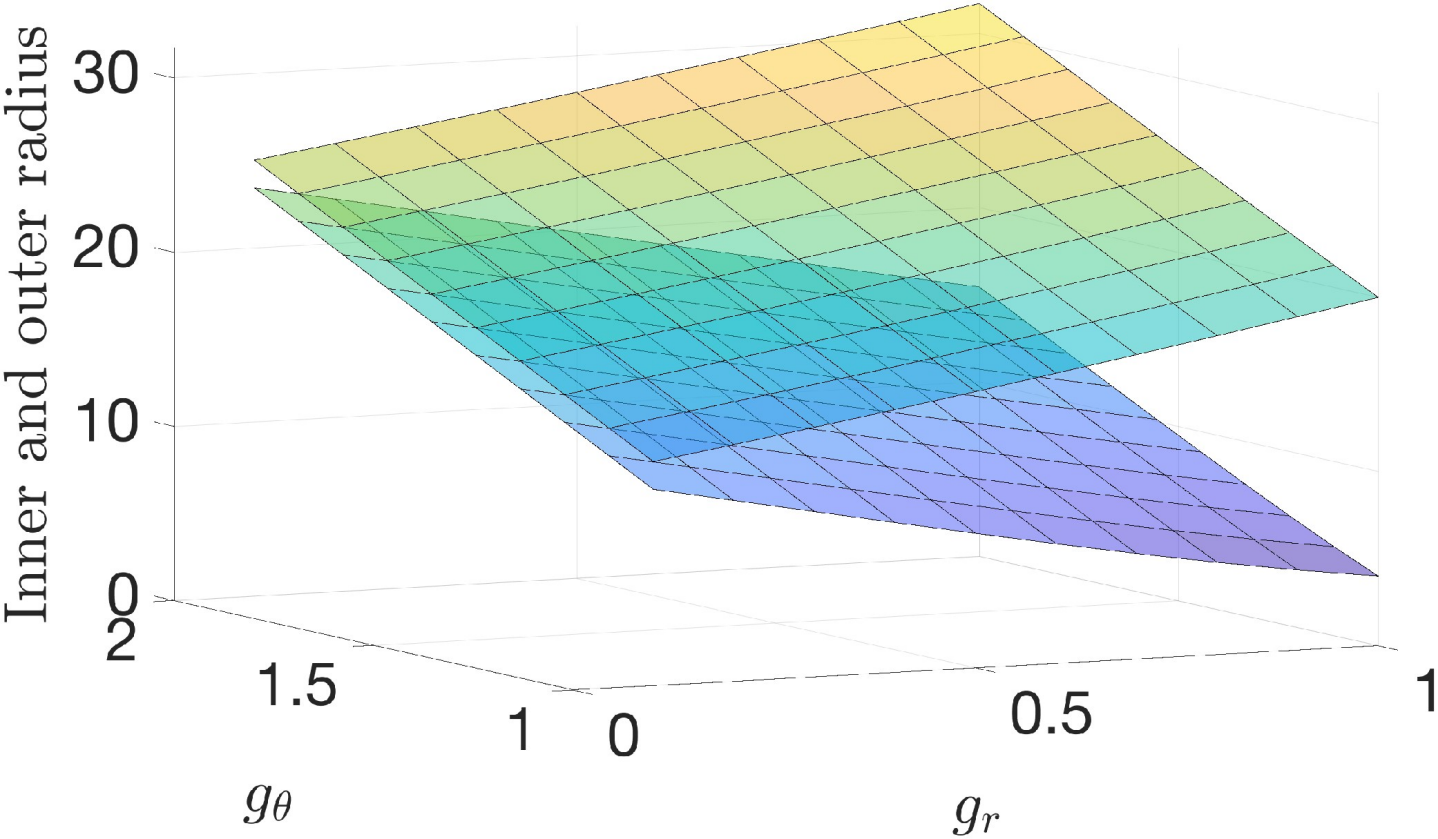
Inner boundary and out boundary radii after growth with different combinations of radial and circumferential growth parameters under *g*_*z*_ = 1. The radial growth parameter *g*_*r*_ is changing over the interval (0.1, 1) for a thinning effect, and *g*_*θ*_ is changing over the interval (1, 2) for a dilation effect; *g*_*z*_ = 1 is kept as a constant. The lower surface shows the inner boundary radius, and the upper surface shows the outer boundary radius. The difference between the upper surface for *r*_4_ and the lower surface for *r*_1_ at the same (*g*_*r*_, *g*_*θ*_) values shows the thickness of the cervical wall.

Fig 8 shows how the radius and thickness of the cervical wall change with various combinations of both *g*_*r*_ and *g*_*θ*_. Where the upper and lower surfaces in the figure are more distant, the cervical wall is thicker. The result demonstrates that thickness reaches its smallest value when *g*_*r*_ reaches its smallest value of 0.1, and *g*_*θ*_ reaches its largest value of 2.0. The thinnest cervical wall is 1.6 mm, only 10% of the original wall thickness of 16 mm. The results show a pattern that smaller radial growth and larger circumferential growth generate a thinner wall. The best dilation effect is also reached for the same pairing of (*g*_*r*_, *g*_*θ*_) values. For other growth parameter pairs, the outer boundary radius may become larger with greater outer layer dilation, but the inner boundary radius is also reduced with smaller inner layer dilation, which is not a good match for smooth birth. Similarly, we can study other combinations, for example, using both *g*_*z*_ < 1 and *g*_*r*_ < 1 for the thinning effect and *g*_*θ*_ > 1 for the dilation effect. For brevity, we skip illustrating these results.

### 3.6 Tissue softening

After studying how growth in different directions affects wall thinning and dilation, we address how tissue softening contributes to the deformation. Tissue stiffness is represented by the shear modulus *μ* and fiber stiffness modulus *γ*. Tissue softening can be indicated by decreasing these two stiffness parameters. The deformation itself is still initiated by growth. We use the growth parameters (*g*_*r*_ = 0.1, *g*_*θ*_ = 2, and *g*_*z*_ = 1), by which an optimal thinning and dilation effect is achieved in Fig 8, to check how different stiffness parameters result in different thinning and dilation effects. The results are shown in Table 6. It illustrates that decreasing the shear modulus *μ* weakens both thinning and dilation but such weakening is only to a small extent. In contrast, when decreasing the fiber stiffness *γ*, both thinning and dilation are strengthened but also only to a slight extent. Such results predict that softening by decreasing the shear modulus cannot facilitate cervical wall thinning and dilation much, and that tissue softening by decreasing fiber stiffness only weakly assists cervical wall thinning and dilation.

**Table 6:** Computational results showing how tissue softening influences thinning and dilation. *g*_*r*_ = 0.1, *g*_*θ*_ = 2, and *g*_*z*_ = 1. Tissue softening is represented by decreasing the shear modulus *μ* or the fiber stiffness *γ*. When *μ* decreases (*γ* = 1000 Pa, the original fiber stiffness), thickness increases slightly and *r*_1_ decreases slightly, i.e., both thinning and dilation are weakened slightly. When *γ* decreases (*μ* = 1650 Pa, the original shear modulus), thickness decreases slightly and *r*_1_ increases slightly, i.e., both thinning and dilation are strengthened slightly.

	*r*_1_ (mm)	*r*_4_ (mm)	Thickness (*r*_4_ − *r*_1_)
*μ* = 1650 Pa	23.4	25.0	1.58
*μ* = 1000 Pa	23.2	24.8	1.60
*μ* = 500 Pa	22.7	24.4	1.62
*μ* = 100 Pa	21.8	23.5	1.69
*γ* = 1000 Pa	23.4	25.0	1.58
*γ* = 500 Pa	23.7	25.3	1.57
*γ* = 100 Pa	24.0	25.6	1.55
*γ* = 10 Pa	24.1	25.6	1.54

## 4 Summary and discussion

For tubular organs, internal tissue growth significantly contributes to organ deformations. Usually growth is employed as an input to generate deformations which are then analyzed for normal physiological alterations or organ malfunctions [28, 29]. In some physiological activities, however, TOs need to acquire necessary deformations to maintain proper functionality under internal growth [18, 25, 26]. Deformation-targeted growth is a novel area for exploration, i.e., using deformations to find growth profiles. Additionally, deformation-targeted growth may be more complex than our initial understanding as both positive and negative growth can simultaneously happen inside the tissue as the TOs develop. Volume increment/decrement may not necessarily mean positive/negative growth in the tissue. Due to the complexity of biological tissue structure, different growth patterns may occur in different dimensions causing more complicated combinations of tissue growth.

Our modeling of the human cervix during pregnancy elucidates how certain types of TODs can be acquired by different types of internal growth. The cervix in pregnancy is observed to gradually demonstrate two important deformations including cervical wall thinning and dilation for smooth birth. No pressure from air or fluid inside the lumen pushes the cervical wall to deform [48], making it different from other TOs such as blood vessels and tracheae. The surrounding ligaments only provide supporting structure for the cervix [56], and thus cannot initiate its deformations. In contrast, hormonally regulated growth [59, 60] is a major factor involved with deformation of the cervix. We employ morphoelasticity with a growth tensor to model the deformation-targeted growth. Growth in three principal morphometric dimensions of the cervix are involved with its deformations (i.e. radial, circumferential, and axial). In the initial simulations, growth in each single direction under no growth occurrence in the other directions is used to show how directionalized growth may facilitate the acquired deformations. While the results reveal that each single-dimensional growth demonstrates unique deformation strength, they also show that growth in any single dimension cannot achieve both thinning and dilation simultaneously. More specifically, the simulations show that negative axial growth (*g*_*z*_ < 1) can assist thinning but not dilation, only positive circumferential growth (*g*_*θ*_ > 1) generates dilation, and negative radial growth (*g*_*r*_ < 1) generates a stronger thinning effect than negative axial growth. Therefore, negative radial growth and positive circumferential growth, under the effect of negative axial growth, are required to generate a significant thinning and dilation effect. Furthermore, we use an assumption of incompressibility to model elastic deformation. While our approach is good for most of pregnancy, towards the end of pregnancy adding a compressibility component may make it more realistic to express part of the volume change due to large changes of biochemical components during pregnancy.

Cervix deformation can also be studied using 3-dimensional finite-element computation techniques. However, one disadvantage of the finite-element technique is that it generally only sustains a very small amount of growth. Large positive or negative growth easily causes the computation to diverge without providing simulation outcomes. The cervix deformation is very large in pregnancy. With only small amounts of growth considered, it is very difficult to show how growth in different principle directions produce distinct thinning and dilation effects. By extracting the main feature of the cervix geometry, we idealize the cervix as an axisymmetric cylinder and consider axisymmetric deformation incurred by growth. The model reduces to an ordinary differential equation problem and can more easily be manipulated by primitive computational approaches. The reduced model accommodates critically large growth effects, and assists us in more easily analyzing how growth patterns in different morphometric dimensions contribute to the thinning and dilation deformation.

The models also ignore irregularities of the cervix geometry. The cervix is not accurately axisymmetric, especially on the top and bottom. Ligaments also surround the cervix as a support structure to the cervix. Further, the cervix is gradually pressed by the uterus above it as the fetus grows. Besides thinning and dilation, the cervix also shortens and forms a “V” shape over its top boundary [61]. Buckling of the inner surface may also happen under growth [62, 63], residual stress can occur in the cervix reference configuration [64], and cervical muscle also contracts during gestation [65]. These factors are not included in the current study. More particularly, residual stress profiles caused by internal growth are significant in modeling deformations of the cervix, and many different residual stress profiles may emerge during pregnancy due to different growth profiles. Similar to blood vessels [66–68], residual stress can mediate the in vivo stress toward homeostatic stress values for the cervix, and can decrease the transmural gradient of cervical wall stress as well. The stress-free reference configuration is obtained usually by cutting the cervix radially to measure the opening angle after the cervix relieves its residual stress [64]. However, it is difficult to obtain human samples to perform such measurements [19]. Thus, it remains elusive how to incorporate residual stress in modeling cervix deformations [10, 69, 70]. Due to this difficulty, residual stress is also ignored in our work and left for future modeling work when more related data are available. Because we use the cervix in early pregnancy as the reference configuration, in which growth has not extensively developed and the lumen pressure from small amount of mucus is very low [19], we expect the residual stress would affect the stress distribution in the cervix but has little effect on the overall deformation as in blood vessels [71].

We mainly studied anisotropic growth in this article as a direction to explore growth complexity. Many other possibilities or their combinations can also be explored, e.g., isotropic growth with inhomogeneous growth over each layer or some layers, isotropic and homogeneous growth with different growth parameters in different layers, and anisotropic growth with inhomogeneous growth in each or some layers. These possibilities can be considered based on future advanced biological understanding of growth profiles in the cervix. When we consider thinning and dilation, it is equally important to consider how extensively the two deformations are achieved based on the fact that the cervix needs to provide a greatly enlarged canal for birth [60]. We are confident that investigation of these possibilities also involves negative and positive growth in different levels verifying growth complexity.

Presently our model provides a qualitative understanding of how required deformation is formed under specified growth. Based on such outcomes, more realistic models accommodating patient-specific geometries can be established to more accurately delineate how the cervix becomes thinned and dilated. Furthermore, the model provides a platform to explore how hormone-regulated biochemical influences, and cell regulations, are associated with growth. By knowing how growth is exhibited in different directions, we can find how biochemical and cellular activities generate corresponding directionalized growth. The growth tensor can be used to derive the surface growth vector over any virtual surface inside the organ to form related growth boundary conditions, comparable to the traditional displacement, force, or pressure boundary conditions. These new growth boundary conditions make the model more straightforward to generate growth-controlled TODs. Our results discover that a mix of positive and negative growth in different directions together contributes to needed deformations. When studying deformation-targeted growth, a uniform growth may not be enough to represent the accurate growth status inside tissues, and more complex growth should be considered. Such complex growth should not be ignored or minimized in importance when studying TODs, and further physiological interpretations and integration with biochemical and cellular activities are needed.
